# Text Processing for Detection of Fungal Ocular Involvement in Critical Care Patients: Cross-Sectional Study

**DOI:** 10.2196/18855

**Published:** 2020-08-14

**Authors:** Sally L Baxter, Adam R Klie, Bharanidharan Radha Saseendrakumar, Gordon Y Ye, Michael Hogarth

**Affiliations:** 1 Viterbi Family Department of Ophthalmology and Shiley Eye Institute University of California San Diego La Jolla, CA United States; 2 Division of Biomedical Informatics Department of Medicine University of California San Diego La Jolla, CA United States; 3 Bioinformatics and Systems Biology University of California San Diego La Jolla, CA United States; 4 Department of Computer Science and Engineering University of California San Diego La Jolla, CA United States

**Keywords:** fungemia, fungal endophthalmitis, fungal ocular involvement, electronic health records, diagnosis codes, regular expressions, natural language processing, unstructured data

## Abstract

**Background:**

Fungal ocular involvement can develop in patients with fungal bloodstream infections and can be vision-threatening. Ocular involvement has become less common in the current era of improved antifungal therapies. Retrospectively determining the prevalence of fungal ocular involvement is important for informing clinical guidelines, such as the need for routine ophthalmologic consultations. However, manual retrospective record review to detect cases is time-consuming.

**Objective:**

This study aimed to determine the prevalence of fungal ocular involvement in a critical care database using both structured and unstructured electronic health record (EHR) data.

**Methods:**

We queried microbiology data from 46,467 critical care patients over 12 years (2000-2012) from the Medical Information Mart for Intensive Care III (MIMIC-III) to identify 265 patients with culture-proven fungemia. For each fungemic patient, demographic data, fungal species present in blood culture, and risk factors for fungemia (eg, presence of indwelling catheters, recent major surgery, diabetes, immunosuppressed status) were ascertained. All structured diagnosis codes and free-text narrative notes associated with each patient’s hospitalization were also extracted. Screening for fungal endophthalmitis was performed using two approaches: (1) by querying a wide array of eye- and vision-related diagnosis codes, and (2) by utilizing a custom regular expression pipeline to identify and collate relevant text matches pertaining to fungal ocular involvement. Both approaches were validated using manual record review. The main outcome measure was the documentation of any fungal ocular involvement.

**Results:**

In total, 265 patients had culture-proven fungemia, with Candida albicans (n=114, 43%) and Candida glabrata (n=74, 28%) being the most common fungal species in blood culture. The in-hospital mortality rate was 121 (46%). In total, 7 patients were identified as having eye- or vision-related diagnosis codes, none of whom had fungal endophthalmitis based on record review. There were 26,830 free-text narrative notes associated with these 265 patients. A regular expression pipeline based on relevant terms yielded possible matches in 683 notes from 108 patients. Subsequent manual record review again demonstrated that no patients had fungal ocular involvement. Therefore, the prevalence of fungal ocular involvement in this cohort was 0%.

**Conclusions:**

MIMIC-III contained no cases of ocular involvement among fungemic patients, consistent with prior studies reporting low rates of ocular involvement in fungemia. This study demonstrates an application of natural language processing to expedite the review of narrative notes. This approach is highly relevant for ophthalmology, where diagnoses are often based on physical examination findings that are documented within clinical notes.

## Introduction

Fungal ocular infection can be highly morbid and vision-threatening. In contrast to bacterial infections, which tend to result from exogenous causes, fungal eye infections more frequently arise endogenously from fungal bloodstream infections [1]. Consequently, they are often diagnosed in inpatient settings, particularly among critical care patients. Although rates of ocular involvement among fungemic patients were reported in early studies to range from 10% to 45% [2-5], most studies in the last two decades have reported rates less than 5% in both adults and children [6-10], a trend attributed to improved systemic antifungal therapies. Although Ghodrasa et al found a slightly higher rate of ocular involvement of 9.2% (22/238) over 5 years, ophthalmic consultation changed management in only 3.8% (9/238) of cases [11]. In short, these studies have shown that a very low percentage of patients with fungemia who are referred for ophthalmological consultation demonstrate ocular involvement.

Prior studies have ascertained positive cases based on manual review of ophthalmic consultation notes, a labor-intensive process, as multiple years of records need to be reviewed given that fungal endophthalmitis is now a relatively rare entity. However, the widespread adoption of electronic health records (EHRs) offers opportunities to facilitate efficient case detection. The most common approach is to utilize structured EHR data in the form of billing or diagnosis codes or other discrete data fields (eg, physiologic measurements, laboratory values, microbiology data). However, structured data comprise a small proportion of the overall data found in EHRs—prior analyses have found that more than 80% of EHR data are unstructured [12]. Natural language processing (NLP) methods have the potential to leverage these unstructured data and are increasingly employed for biomedical applications [13-16]. One impactful application of NLP is information extraction from free-text clinical notes [17-19], which is especially relevant for ophthalmology, where many diagnoses are based on physical examination findings described in free-text notes rather than structured data such as laboratory values. NLP has been applied to extract data on visual acuity [20] and intracameral antibiotic injections and posterior capsular rupture [21]. It has also been used to extract surgical laterality and intraocular lens implant power and model information [22], glaucoma-related characteristics [23], measurements of epithelial defects and stromal infiltrates in microbial keratitis [24], as well as identification of herpes zoster ophthalmicus [25] and pseudoexfoliation [26].

In this study, we evaluated the prevalence of fungal ocular involvement in the Medical Information Mart for Intensive Care III (MIMIC-III), a cohort of over 46,000 critical care patients over 12 years. Our objective was to detect cases of any fungal ocular involvement (including fungal endophthalmitis, vitritis, and chorioretinitis). In addition to the traditional approach of using manual record review of all patients, we also used queries based on discrete diagnosis codes and developed a novel NLP-based approach for rapid detection of relevant free-text clinical notes related to the diagnosis.

## Methods

### Study Population

The study population consisted of all patients with fungemia in MIMIC-III, a database of patients admitted to critical care units at the Beth Israel Deaconess Medical Center, a tertiary care hospital in Boston, Massachusetts [27]. It includes deidentified data from over 46,000 critical care patients (adults and neonates) from 2001-2012. In addition to structured data elements such as admission information, demographics, laboratory values, diagnosis codes, intervention/procedure codes, microbiology data, medications, and physiologic measurements, MIMIC-III also includes deidentified free-text clinical notes such as provider admission and progress notes, discharge summaries, consultation notes, and free text reports of electrocardiogram and imaging studies [27]. All authors underwent appropriate Health Insurance Portability and Accountability Act (HIPAA) and Collaborative Institutional Training Initiative (CITI) research ethics training and completed the required data use agreements before accessing any data from MIMIC-III. The University of California San Diego Institutional Review Board (IRB) ruled that approval was not required for this study, as it qualified as non-human subjects research. Consent was not obtained, given that participants were not identifiable. The research adhered to the tenets of the Declaration of Helsinki and conformed with all country, federal, and state laws.

The population of interest consisted of all patients with fungal bloodstream infections, as these individuals would typically be referred for ophthalmological evaluation according to current guidelines from the Infectious Disease Society of America and standard practice patterns [28]. We identified these patients via a structured query language (SQL) query of the “MICROBIOLOGY EVENTS” table in MIMIC-III, selecting for all patients with documented positive blood cultures containing fungal organisms. All fungal species were included. This query yielded 265 patients.

### Case Detection via Processing of Structured Data

We defined the outcome of interest as the development of fungal ocular involvement during the documented hospitalization involving culture-positive fungemia. First, we screened for fungal ocular involvement using structured data consisting of International Classification of Disease version 9 (ICD-9) diagnosis codes. As the data in MIMIC-III spanned 2001-2012, there were no ICD-10 codes, which were not available until 2015. We obtained ICD-9 codes by querying the “DIAGNOSES_ICD” table in MIMIC-III, which entails hospital-assigned diagnoses for each patient during each hospitalization and joining the codes with the “D_ICD_DIAGNOSES” table, which is a dictionary of ICD-9 codes and descriptions. To account for known limitations of billing and diagnosis codes such as incomplete coding by clinicians and insufficient granularity of codes [29], we included a broad range of diagnosis codes related to endophthalmitis, retinal disorders, chorioretinal inflammations, vitreous disorders, visual disturbances, and blindness and low vision (see Multimedia Appendix 1 for specific ICD-9 codes). Records for patients with relevant diagnosis codes were then reviewed to determine whether there was any documentation of fungal ocular involvement.

### Case Detection via Natural Language Processing of Unstructured Data

We also screened for cases of fungal ocular involvement by analyzing unstructured data consisting of free-text notes available in the MIMIC-III database. Given the large volume of clinical notes for the study population (26,830 total notes for 265 patients), we used NLP methods to facilitate note review and identification of cases. We used a regular expression pipeline based on string matching to extract relevant text strings from notes surrounding the term(s) specified in the regular expression. Regular expressions are strings written in a standardized computational grammar that can be used to identify patterns in free text; they offer an effective strategy for many NLP problems involving pattern matching [30]. Regular expressions have been used in several biomedical contexts to extract information such as conditions and medications from free text [31-34]. Here, we used a variety of terms embedded in regular expressions, ranging from specific single phrases (eg, “fungal endophthalmitis”) to multiterm expressions (eg, “ophth|opth|eye|ocul|retina|fundus|dilat|endophthalmitis| chorioretinitis|vitritis”). We used a string-matching approach for its simplicity and straightforward implementation. Common misspellings, such as “opth-” instead of “ophth-,” were accounted for in the search. In a strategy similar to the one used for the diagnosis code queries, a broad array of terms were included in the regular expressions in order to err on the side of sensitivity, improving the likelihood of case detection, particularly because fungal ocular involvement is a rare condition. We did not include “Candida” in the regular expressions because this term had been removed from the MIMIC-III database as part of de-identification protocols since “Candida” can be a female first name. Therefore, any mentions of “Candida endophthalmitis” would be obscured as “[**Female First Name (un)**] endophthalmitis.” However, because terms such as “endophthalmitis,” “vitritis,” “chorioretinitis,” “ocular involvement,” and other terms related to the eye and fungal infections were included, our strategy was designed to maximize the likelihood of identifying those cases without explicitly including “Candida” in the regular expressions.

We recorded the number of text strings resulting from each pattern-matching script as well as the run time for each script to produce an output. Text strings and subsequently, full clinical notes were manually reviewed to determine whether fungal ocular involvement was documented. The Python package *re* was used as the regular expression interpreter [35]. Open-source coding details are available on GitHub [36].

### Manual Record Review

A full manual record review was performed for all 265 fungemic patients in this cohort by a practicing ophthalmologist (SB). This involved review of all structured (eg, diagnosis codes, microbiology values) and unstructured (eg, notes) data for each patient to identify any mention of fungal ocular involvement. The rationale for this was to establish a “gold standard” using a traditional approach and to ascertain whether there were any additional cases of fungal ocular involvement that may not have been identified by querying diagnosis codes or regular expression-based searches.

### Other Patient Characteristics

In addition to the outcome of fungal ocular involvement for each patient, we also recorded demographic data, fungal species present in blood culture, and known risk factors for fungemia such as the presence of indwelling catheters, recent major surgery, diabetes, immunosuppression (either from underlying conditions or induced medically by immune-suppressing medications such as chemotherapy), history of intravenous drug use, and hyperalimentation. We obtained data regarding these various factors via SQL queries of the relevant tables in MIMIC-III based on current procedural terminology (CPT) events and ICD-9 codes for diagnoses and procedures. Definitions for risk factors that we used in the queries are detailed in Multimedia Appendix 2. Data were processed and analyzed using R [37].

## Results

**Figure 1:**
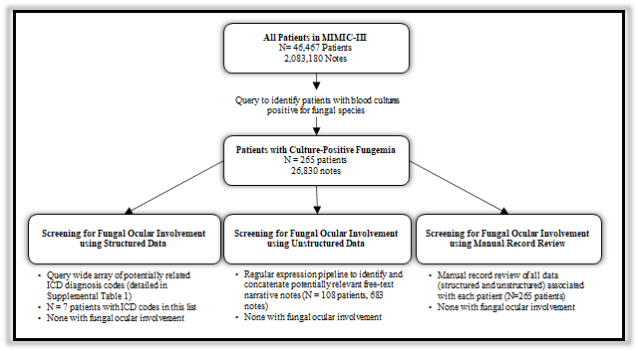
Screening for fungal ocular involvement using approaches leveraging structured and unstructured data within the Medical Information Mart for Intensive Care III (MIMIC-III). SQL: Structured Query Language; ICD: International Classification of Disease.

Figure 1 depicts an overview of the study methodology and results. In total, 265 patients in the MIMIC-III database had positive blood cultures containing fungal species. The mean (SD) age was 62.3 (16.8) years. A majority (199/265, 75%) self-identified as white, and there were slightly more males (154/265, 58%) than females (Table 1). The mean (SD) length of stay was 24.6 (27) days. The in-hospital mortality rate for these fungemic patients was high, with 121 (41%) who died during hospitalization, reflecting the severity of illness. The most common fungal species found on blood cultures were *Candida albicans* (114, 43%), *Candida glabrata* (74, 28%), *Candida parapsilosis* (32, 12%), and *Candida tropicalis* (21, 8%).

Over one-half of fungemic patients had recent major surgery (n=148, 55.8%), over one-third had indwelling central catheters (n=96, 36.2%), and over one-quarter carried a diagnosis of diabetes (n=69, 26.0%; Table 1). Other known risk factors for fungemia, such as cancer, immunosuppressed status from chemotherapy or steroids, intravenous drug use, and hyperalimentation, were relatively uncommon (all <10%) in this cohort.

**Table 1:** Characteristics of patients with fungemia in MIMIC-III (N=265).

Characteristics		Value, n (%)
**Gender**		
	Female	111 (41.9)
	Male	154 (58.1)
**Ethnicity**		
	White	199 (75.1)
	Asian	6 (2.3)
	Black/African American	22 (8.3)
	Hispanic or Latino	5 (1.9)
	Other	3 (1.1)
	Patient declined or unknown	30 (11.3)
**Risk factors for fungemia**		
	Indwelling central catheter	96 (36.2)
	Diabetes	69 (26.0)
	Cancer	19 (7.2)
	Chemotherapy	4 (1.5)
	Steroids	7 (2.6)
	Intravenous drug use	11 (4.2)
	Bone marrow transplant	0 (0)
	Hyperalimentation	2 (0.8)
	Surgery	148 (55.8)

### Screening for Fungal Ocular Involvement Using Structured Data

A query of the MIMIC-III diagnosis tables among this cohort of fungemic patients using a broad range of eye- and vision-related ICD-9 codes (Multimedia Appendix 1) yielded 7 unique patients. For these patients, there were 1079 total associated clinical notes in the database. Examination of these records revealed that none had fungal ocular involvement. For example, one patient had been coded as having unilateral vision loss, but based on a review of her records, this was attributed to ischemic optic neuritis, not a fungal infection. Another patient endorsed blurry vision and poor visual acuity following a transfer out of the intensive care unit, prompting dilated fundus examination—there were diabetic proliferative changes noted but no evidence of fungal ocular involvement. Other reasons for ICD-9 codes related to vision changes among the fungemic patients in the cohort included occipital lobe stroke and posterior subcapsular cataracts. Out of all fungemic patients with ICD-9 codes related to vision changes, none were found to have fungal ocular involvement as the cause of their visual symptoms.

### Screening for Fungal Ocular Involvement Using Unstructured Data

To leverage the free-text narrative notes in this database, we used a range of regular expressions to identify potential cases of fungal ocular involvement. These regular expressions formed the main component of pattern-matching scripts that returned the matches and surrounding text. First, we used algorithms centered on single phrases, such as “fungal endophthalmitis,” “fungal endopthalmitis” (accounting for common misspelling), and “fungal ocular involvement.” “Fungal endophthalmitis” yielded 3 notes, “fungal endophthalmitis” yielded one note, and “fungal ocular involvement” yielded no notes.

**Figure 2:**
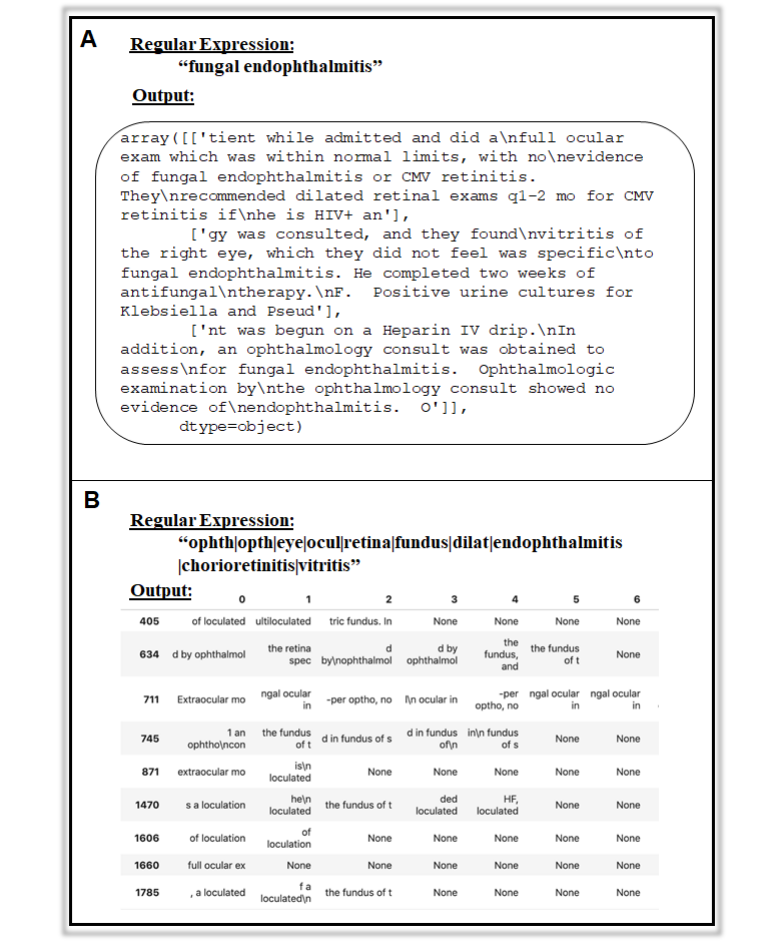
Examples of output from regular expressions screening for fungal endophthalmitis. (A) Output with 100 characters on each side from 3 patients with “fungal endophthalmitis” mentioned. (B) Output with 5 characters on each side from a multi-term regular expression. Rows indicate patients, and columns indicate notes. In this example illustration, only the first 6 notes are displayed for a sample of patients. “None” indicates no matches from the regular expression were found in the note.

Next, we used a broader range of terms related not only to potential findings from fungal ocular involvement (eg, endophthalmitis, chorioretinitis, vitritis) but also to general eye examinations (eg, dilation, fundus, eye). This approach yielded 4000 notes from 255 patients. However, many mentions of “eye” and “dilat” had no relationship to fungal ocular involvement as these were very general terms. To improve specificity, we removed “eye” and “dilat” from the regular expression, resulting in 683 notes from 180 patients. Examples of the output from the regular expressions are depicted in Figure 2. Our strategy included not only the identification of relevant strings but also the concatenation of all relevant text strings together for each patient to facilitate a subsequent review. Based on a manual review of the relevant text strings identified by the algorithm in these notes, followed by a manual review of the full-length notes, we still did not identify a single case of fungal endophthalmitis or any variation of fungal ocular involvement.

### Manual Record Review

A full manual record review was conducted for all 265 patients, encompassing a full-text review of all 26,830 notes as well as all structured data. There was no evidence of fungal ocular involvement for any patient in the cohort, even after manual record review.

## Discussion

A major finding from this study was there was not a single case of fungal ocular involvement identified among culture-proven fungemic patients in the MIMIC-III database, which encompasses >50,000 hospital admissions for >46,000 patients between 2000 and 2012. This result is consistent with several other studies where rates of fungal ocular involvement were very low, generally <5% in studies conducted over the last two decades [6-10]. Most of those studies examined periods of 5 years or less, whereas here we did not detect a single case over 12 years. These patients demonstrated several risk factors associated with fungemia, as one-half had recent major surgery (n=148, 55.8%), over one-third had indwelling central catheters (n=96, 36.2%), and over one-quarter carried a diagnosis of diabetes (n=69, 26.0%; Table 1). Indicative of the severity of infection and illness, this cohort had a high mortality rate, with over 40% of patients dying during the documented hospitalization. Therefore, there is no evidence to suggest that the lack of fungal ocular involvement was because this cohort was healthier or lower risk than those in prior studies.

Some have argued that targeted ophthalmic screening of patients with fungemia, rather than the universal screening that is currently recommended [28], may be a better use of personnel and financial resources, given the low rates of fungal ocular involvement and the rarity of changes in clinical management based on ophthalmic consultation [7,8,11,38,39]. However, prior studies have not rigorously described criteria for guiding targeted screening. Although there appears to be a consensus that nonverbal patients who are unable to communicate visual symptoms should be screened, there have not been rigorous reports of further risk stratification. Presumably, one reason for this is that the incidence of fungal ocular involvement is so low that achieving sufficient numbers for appropriately powered statistical models is difficult. With increasing efforts toward building multicenter clinical data warehouses, where data from multiple centers can be aggregated and analyzed together, there may be increased opportunities in the future to improve risk stratification.

However, the increasing volume of data in the current era of EHRs presents challenges. For example, in this cohort, there were almost 27,000 notes associated with fungemic patients. To address this, we developed a regular expression pipeline as an NLP-based approach to facilitate note review. NLP has been used to abstract findings from radiology and pathology reports [40-44], for identifying phenotypes from narrative notes [45-48], and specifically for ophthalmic data extraction such as visual acuity and surgical complications [20-22]. NLP has been shown to enhance case detection compared to structured diagnosis codes alone, for example, for identifying cases of pseudoexfoliation syndrome [26] and herpes zoster ophthalmicus [25]. Structured diagnosis codes have known limitations such as incomplete or inaccurate coding, insufficient granularity, and the fact that clinicians may not code for every condition at every encounter [29]. In this specific context, diagnosis codes likely have low sensitivity for fungal ocular involvement because ophthalmologists who examine patients serve in consultant roles rather than as primary providers. Therefore coding is typically performed by non-ophthalmologists who may not be familiar with eye-related diagnoses.

Our regular expression pipeline efficiently identified relevant text strings, extracted the associated notes, and subsequently collated them together for further review. Run times were short, at less than 3.5 seconds, much faster than manually combing through 27,000 notes, which took several weeks. We used simple regular expressions based on string-matching that were straightforward to implement. These were run on a standard local machine without requiring a high-performance computing infrastructure. We are unaware of previous reports of using NLP-based information extraction to identify cases of fungal ocular involvement and could not find any reference to this type of application in a PubMed search. Therefore, this approach represents a novel efficient “search” of a large volume and variety of critical care notes to identify the most relevant notes related to this diagnosis. Improving search functionalities will be crucial as EHRs generate an increasing volume of unstructured data requiring analysis.

Several important points arise when considering the issues of sensitivity and specificity of the structured versus unstructured approaches. Because we knew fungal ocular involvement to be a relatively rare condition based on prior studies, we erred on the side of sensitivity and cast a wide net in terms of included diagnosis codes as well as regular expressions. For this reason, we also did not include negation terms in the regular expressions in order to avoid inadvertently excluding any records with pertinent eye findings. At first glance, the structured diagnosis code query may seem to have been “better” than the unstructured NLP approach since it identified fewer patients and therefore appeared more specific and more efficient. However, in several prior studies, using diagnosis codes alone yielded fewer positive cases, resulting in under-detection and decreased sensitivity [25,26]. In this study, we could not assess the relative sensitivity and specificity of each approach, given that there were zero cases of fungal ocular involvement. However, because we manually reviewed the remaining notes that were not returned by the search, we were confident that these search terms did not “miss” any positive cases. Future studies of cohorts that include positive cases would benefit from comparing these metrics across different approaches that leverage structured data, unstructured data, or both. In addition, refining search strategies to include more nuanced approaches such as negation terms would also be relevant in future work.

We considered the possibility that there may have been true cases of fungal ocular involvement that were missed due to one or more of the following: (1) we did not include the appropriate diagnosis codes in our query, (2) we did not include the appropriate terms in our regular expressions, or (3) rare conditions may have been removed from the database as part of de-identification protocols. To address the first two possibilities, we performed a full manual record review of all data (structured and unstructured) for these 265 fungemic patients. There were still no cases of fungal ocular involvement. To address the possibility of removal for de-identification purposes, we contacted the principal investigators of the MIMIC-III database, who confirmed that rare diseases were not excluded from the database as part of any de-identification procedures. Therefore, we are confident that our findings reflect the true prevalence of fungal ocular involvement among patients with culture-proven fungemia in this database.

This study had some limitations. First, some of these patients may have developed fungal ocular involvement in the regular wards or the outpatient setting after being downgraded or discharged from critical care units, and this would not have been reflected in the inpatient critical care records analyzed. In addition, this database was based on a single academic medical center, so it is not clear whether these findings would generalize to other settings. However, fungemic patients tend to have significant co-morbidities and are, therefore, often treated at the tertiary care level. Despite being limited to a single center, the 12-year longitudinal period of study encompassed in the database allowed for a large number of patients to be analyzed. There may have been patients with highly suspected fungemia and eye findings presumed to be fungal endophthalmitis. However, we did not include them in this analysis due to the difficulty of precisely defining “highly suspected fungemia” using either diagnosis codes or text. By restricting the analysis to patients with culture-proven fungemia, we increased certainty around the denominator in the prevalence calculation. Finally, only five patients had ICU providers who had copied and pasted ophthalmic exam findings verbatim into their notes. For the remaining patients, ICU notes contained summaries stating the ophthalmology consultation did not show any evidence of ocular involvement, but not a direct recapitulation of ophthalmic exam findings. Some patients may have had positive eye exam findings that were not captured in ICU provider notes, which is a limitation of using this particular data source. However, in clinical practice, typically, the detection of fungal ocular involvement is a rare and notable event such that the likelihood of an ICU provider not documenting a positive case would be low.

In summary, we demonstrated an application of NLP-based methods to a large-scale clinical database to gain insights about the prevalence of fungal ocular involvement. As clinical research increasingly includes unstructured narrative notes in addition to structured data, NLP will play a growing role in phenotyping. This approach will be critical for ophthalmological entities where details and descriptions are often embedded within notes rather than within structured data fields.

## Appendix

Multimedia Appendix 1 Structured diagnosis codes used to screen for fungal ocular involvement. ICD-9 = International Classification of Diseases, version 9.

Multimedia Appendix 2 Definitions of features extracted from MIMIC-III regarding patient characteristics and risk factors for fungemia.

## Abbreviations

CITI: Collaborative Institutional Training Initiative
CPT: Current Procedural Terminology
EHR: electronic health record
HIPAA: Health Insurance Portability and Accountability Act
ICD: International Classification of Disease
IRB: Institutional Review Board
MIMIC-III: Medical Information Mart for Intensive Care III
NLP: natural language processing
SQL: Structured Query Language
