# Supplementary material for: Text Processing for Detection of Fungal Ocular Involvement in Critical Care Patients: Cross-Sectional Study
Source: J Med Internet Res. 2020 Aug 14;22(8):e18855. doi: 10.2196/18855 (PMC7455861; doi:10.2196/18855)
Supplement: Multimedia Appendix 1 [file jmir_v22i8e18855_app1.docx]

**Multimedia Appendix 1. Structured diagnosis codes used to screen for fungal ocular involvement.** ICD-9 = International Classification of Diseases, version 9.

| **ICD-9 Code** | **Description** |
| --- | --- |
| 360.00 | Purulent endophthalmitis, unspecified |
| 360.01 | Acute endophthalmitis |
| 360.02 | Panophthalmitis |
| 360.04 | Vitreous abscess |
| 360.19 | Other endophthalmitis |
| 360.9 | Unspecified disorder of globe |
| 362.8 | Other retinal disorders |
| 362.9 | Unspecified retinal disorder |
| 363^a^ | Chorioretinal inflammations, scars, and other disorders of the choroid |
| 368.1^a^ | Subjective visual disturbances |
| 368.4^a^ | Visual field defects |
| 368.8 | Other specified visual disturbance |
| 368.9 | Unspecified visual disturbance |
| 369^a^ | Blindness and low vision |
| 379.2^a^ | Disorders of vitreous body |
| 379.9^a^ | Unspecified disorder of eye and adnexa |

^a^All diagnoses within the parent code were also specified in the query.
