# Supplementary material for: Text Processing for Detection of Fungal Ocular Involvement in Critical Care Patients: Cross-Sectional Study
Source: J Med Internet Res. 2020 Aug 14;22(8):e18855. doi: 10.2196/18855 (PMC7455861; doi:10.2196/18855)
Supplement: Multimedia Appendix 2 [file jmir_v22i8e18855_app2.docx]

**Multimedia Appendix 2. Definitions of features extracted from MIMIC-III regarding patient characteristics and risk factors for fungemia.**

| ***Feature*** | ***Definition*** |
| --- | --- |
| Age | DOB (date of birth) subtracted from CHARTDATE |
| Gender | Directly derived from PATIENTS table |
| Diabetes | ICD-9 codes: 25000:25900 |
| Fungal Species | Organism identified in blood cultures from MICROBIOLOGY EVENTS table |
| Indwelling central catheters | CPT codes: 36620, 36555, 36569, 36555:36571, 36575:36576, 36578, 36580:36585, 36589:36596 |
| Major Surgery |  |
| Wound exploration/trauma | CPT codes: 20100:20103 |
| Surgical procedures on the musculoskeletal system | CPT codes: 20150:20251, 20500:20697, 20802:20838, 20900:20939, 20950:20999 |
| Surgical procedures on the neck and thorax | CPT codes: 21501:21899 |
| Surgical procedures on the spine | CPT codes: 22010:22899 |
| Surgical procedures on the abdomen, peritoneum, and omentum | CPT codes:  22900:22999, 49000:49999 |
| Surgical procedures on the pelvis and hip joint | CPT codes: 26990:27299 |
| Surgical procedures on the trachea and bronchi, lungs and pleura | CPT codes: 31600:31899, 32035:32999 |
| Surgical procedures on the cardiovascular system | CPT codes: 33010:37799 |
| Surgical procedures on the hemic and lymphatic systems | CPT codes: 38100:38999 |
| Surgical procedures on the mediastinum and diaphragm | CPT codes: 39000:39599 |
| Surgical procedures on the stomach | CPT codes:  43500:43999 |
| Surgical procedures on the intestines | CPT codes: 44005:44799, 44800:44899, 44900:44979, 45000:45999 |
| Surgical procedures on the liver, biliary tract, and pancreas | CPT codes: 47000:47399, 47400:47999, 48000:48999 |
| Surgical procedures on the kidney | CPT codes:  50010:50593 |
| Surgical procedures on the nervous system | CPT codes: 61000:62258, 62263:63746 |
| Cesarean Delivery Procedures | CPT codes: 59510:59525 |
| Immunosuppression |  |
| Cancer | ICD-9 codes: 14000:23970, 2733, 27789 |
| Chemotherapy | ICD-9 codes: 9925, B5810, V6620, V6700, V5811, V8741, 5497, 9925, 3491, 0392, 9649, 0010, 8607  CPT codes: 96400, 96408:96425, 96520, 96530 |
| Long-term broad spectrum antibiotics | ICD-9 codes: V5862 |
| Steroids | ICD-9 codes: V5865, 9923 |
| Bone Marrow Transplant | ICD-9 codes: V4281, 4100:4109 |
| Intravenous Drug Use | ICD-9 codes: E8500, E8502, 30400:30403, 30470:30473, 30550:30553, 96501, 96509, 30421:30423, 30560:30563; 97081, 30441:30443, 30571:30573, 96972, 30540:30543, 30460:30463, 30480:30483, 30490:30493, 30590:30593, 64833, 64834 |
| Hyperalimentation | CPT codes: 99601, 99602  ICD-9 codes: V5869, 2788, 9915 |
